# Impact of Mental Health Literacy on Improving Quality of Life Among Adolescents in Barcelona

**DOI:** 10.3390/children12020235

**Published:** 2025-02-15

**Authors:** Isaac Daniel Amado-Rodríguez, Rocio Casañas, Jaume Juan-Parra, Juan Francisco Roldan-Merino, Lluís Lalucat-Jo, Mª Isabel Fernandez-San-Martín

**Affiliations:** 1 PhD Program in Biomedical Research Methodology, Public Health Autonomous University of Barcelona, 08193 Barcelona, Spain; 2 Department of Nursing, University of Girona, 17003 Girona, Spain; rocio.casanas@udg.edu; 3 Unitat de Suport a la Recerca Barcelona Ciutat, Idiap JordiGol, 08025 Barcelona, Spain; jjuan@idiapjordigol.org; 4 Sant Joan de Déu Campus Docent—Private Foundation, School of Nursing, 08034 Barcelona, Spain; jroldan@santjoandedeu.edu.es; 5 Associació Centre d’Higiene Mental Les Corts, Grup CHM Mental Health, 08029 Barcelona, Spain; lluis.lalucat@chmcorts.com; 6 Unitat Docent Multiprofessional Atención Primaria Barcelona, Institut Català de la Salut, 08025 Barcelona, Spain; mifsanmartin.bcn.ics@gencat.cat

**Keywords:** mental health literacy, adolescent, quality of life, mental well-being, EuroQoL

## Abstract

**Background/Objectives**: We aim to assess the effect of the Espaijove.net mental health literacy program on adolescents’ quality of life (QOL). Additionally, we aim to describe their QOL and mental well-being. **Methods**: A multicenter, randomized, controlled trial was conducted, with pre- and post-intervention assessments and 6- and 12-month follow-ups. A total of 1032 students aged 13–14 from 18 schools in Barcelona participated in one of the three following mental health literacy (MHL) programs or were placed in a control group (CG): (1) a 1 h awareness session (G1h); (2) a 6 h MHL program (G6h); (3) a 7 h MHL program with stigma reduction (G7h). Measures: (1) Mental well-being: Strengths and Difficulties Questionnaire (SDQ); (2) QOL: EuroQol 5D-5L with its two parts: the EuroQol 5D-5L Index (0–1) and EuroQol 5D-5L visual analog scale (EQ-VAS) (0–100). Analyses were conducted on an intention-to-treat basis, using data imputation methods for missing data. Intervention effects were assessed using multilevel models. **Results**: Baseline EQ-VAS and EQ-5D-5L index scores were 77.84 (CI = 76.77–78.91) and 0.91 (CI = 0.90–0.92), respectively. Boys reported higher QOL and SDQ scores (*p* < 0.001), whereas participants of foreign nationality showed lower scores in QOL (EQ-VAS; *p* = 0.039) and mental well-being (*p* < 0.001). Post-intervention, all groups (intervention and control), except G6h, showed QOL improvements. However, in the 6-month follow-up, the CG outperformed the other groups. At 12 months, G7h achieved the highest EQ-VAS scores compared to the other groups. **Conclusions**: MHL-based interventions improved short-term QOL but failed to sustain these improvements over time. Groups with lower QOL and SDQ scores included girls and adolescents of foreign nationality.

## 1. Introduction

Quality of life (QOL), mental health, and mental well-being are interrelated concepts. The World Health Organization defines QOL as “the individual’s perception of their position in life in the context of the culture and value systems in which they live and in relation to their goals, expectations, standards, and concerns” [1]. This is an idea that encompasses different aspects of human comfort and individuals’ degrees of satisfaction with life.

In Spain, a 2018 study found that adolescents aged between 11 and 18 years had a medium (61.8%) and high (37.4%) health-related QOL, with only 0.8% reporting a low one. Boys scored higher than girls, and a higher degree of QOL was more frequent among subjects whose families had a stronger economic status. Some psychological distress in the previous six months was observed in 53.3%, whilst 10.1% considered their health to be acceptable [2]. Another Spanish study, the FRESC in Barcelona, reported that 10.1% of adolescents considered their perceived health to be normal or bad [3]; moreover, 38.6% of girls and 20.3% of boys reported suffering emotional distress. Both studies concurred that adolescents from families with a greater economic status tended to present better QOL than those with fewer resources [2,3].

One of the most influential factors in QOL is mental health [4]. In comparison with the general population, individuals with mental health issues live a mean of 10 to 15 years fewer [5] and may present a greater probability of experiencing worse mental well-being [6]. Mental health disorders usually manifest at an early age between 12 and 25 years [5,7]. Nevertheless, currently, 34.6% of these conditions have been observed to initiate before the age of 14 years, and 62.5% emerge before the age of 25 [6].

Health literacy is a predictive indicator of QOL in both adults and adolescents [8], and its improvement leads to better QOL [9,10]. Strategies have been introduced to ameliorate a population’s mental health. One of these tools is mental health literacy (MHL), which was defined by Jorm et al. as the set of “knowledge and beliefs about mental health problems that aid in their recognition, management, and prevention” [11]. Other experts have defined it as a tool that facilitates an understanding of how to achieve and maintain optimal mental health, identify mental disorders and their treatments, decrease associated stigma, and promote more effective help-seeking [12].

An optimal MHL is crucial for improving well-being and mental health at both collective and individual levels [13,14,15,16]. MHL-based interventions in adolescents have led to an increase in mental health knowledge and, to a lesser extent, a decrease in stigma [17,18]. They have also affected help-seeking [19]. Few studies have examined the effect of these interventions on adolescent QOL. Nevertheless, psychological interventions and those based on the promotion of good mental health have been shown to improve QOL in adults presenting some pathology [20,21].

We carried out a clinical trial to evaluate the short- and long-term effects of a universal MHL intervention, EspaiJove.net, on improving the knowledge of mental health, help-seeking, and stigma reduction in the adolescent population [22]. Our findings showed that the different levels of interventions in the EspaiJove.net program did not appear to be effective with respect to MHL, stigma reduction, or help-seeking behavior. Nevertheless, while contact with an individual who had experienced a mental illness did not decrease stigma, it did increase knowledge about mental health [22].

In this study, we present the results of the QOL and mental health variables of the adolescent participants at baseline and the impact of the EspaiJove.net program on their QOL.

Our objective was to assess the effectiveness of the three intervention modalities of the EspaiJove.net MHL program on adolescents’ QOL in the short (post-intervention) and long term (follow-up at 6 and 12 months). A secondary objective was to assess their degree of QOL and mental well-being with respect to their sociodemographic and socioeconomic variables.

## 2. Materials and Methods

### 2.1. Design

A controlled, randomized, multicenter trial was carried out in 18 schools in Barcelona, Spain. The trial was based on four levels of intervention. The study protocol has been described by Casañas et al. [23]. It was designed and carried out according to the CONSORT 2010 standards for randomized, controlled trials for conglomerates [24] and registered at Clinical.Trial.gov (NCT03215654; dated 7 December 2017).

### 2.2. Participants

Inclusion criteria were adolescent students aged 13–14 years, those enrolled in their 3rd year of E.S.O (compulsory secondary education) in a public or private school in the city of Barcelona, and those who accepted an informed consent agreement. Those attending special education schools and schools where the official language was not Spanish/Catalan were excluded. Individuals who did not understand Spanish/Catalan, who had special educational needs, and who had previously participated in an EspaiJove.net workshop were also excluded.

### 2.3. Sample Size

A power analysis was carried out to determine the appropriate sample size based on the study by Dams et al. [25]. Considering an alpha risk of 0.05 and a statistical power of >0.8 in a bilateral contrast, a minimum of 234 subjects for each group were required to detect a statistically significant minimum difference of 0.09 units between the pairs of groups (assuming there were four). We estimated a common standard deviation (SD) of 0.25 and a follow-up drop-out rate of 20%. The sample was increased to 1298 following the cluster design described by Casañas et al. [22].

### 2.4. Recruitment Process

Recruitment was carried out from September 2016 to December 2017. An informative email was sent to all the schools in Barcelona, of which only 18 agreed to take part. Once participation had been accepted, the 18 schools received letters of consent, which they were then responsible for sending to the students’ parents/guardians.

All participating students signed informed consent forms to participate in the study. The data of minors were protected through an anonymous code. The Ethics Committee of the Fundació Catalana d’Hospitals approved the study (CEIC 15/33). The selected centers were randomly distributed between an intervention group and a control group. Randomization was carried out by clusters (EC), stratified according to the number of classes in the school (less than 5 classes and more than 5 classes). Cluster randomization (educational institutions) was performed with a computer program, using a 1:1:1:1:1 distribution for the cluster. This process was carried out by a statistician outside the research team.

### 2.5. Procedure

Data were gathered at four intervals: Pre-intervention questionnaires were administered 2 weeks before the intervention for all students (control group (CG) and intervention groups (IG)). Two weeks after the end of the intervention (one month after baseline), an assessment was conducted for the IG and one month after baseline for the CG. Finally, all groups were re-evaluated at 6- and 12-month post-interventions.

### 2.6. Intervention

The three intervention modalities have been described in the study protocol (Casañas et al., 2018) [23] and in an article regarding the evaluation of their effectiveness [22]. They are listed in the following:

(1) Sensitivity program (G1h): This one-hour program introduces the concepts of mental health and mental disorders (emotional management) to the participants.
(2) Mental health literacy program (G6h): This 6 h MHL-based module includes the following: (a) concept of mental health and mental disorders; (b) healthy behaviors and risk factors in mental health; (c) interpersonal skills and antisocial behavior, including bullying and cyberbullying: (d) anxiety disorders, depression, self-harm, and suicidal behavior; (e) eating and behavioral disorders; (f) substance abuse and psychotic disorders.
(3) MHL program and stigma reduction (G7h): A six-hour MHL program plus one hour of stigma reduction by means of participants coming into an interaction with a person with a mental problem.
(4) The CG did not receive any intervention.

### 2.7. Variables and Measuring Tools

Data regarding the participants’ sociodemographic variables (gender, age, and nationality) and place of residence (municipality/district/neighborhood) were gathered. Socioeconomic status was measured with the Household Disposable Income Per Capita index (HDIpc). This tool defines the volume of resources (in EUR) that households have available for consumption or savings in a territory [26].

Each participant was assigned the corresponding HDIpc for their neighborhood if they lived in Barcelona. Those residing outside of the city were allocated the HDIpc of their municipality [27,28]. There were five HDIpc categories: high (>1.3), medium–high (1.1–1.29), medium (0.9–1.09), medium–low (0.7–0.89), and low (<0.7).

Quality of Life: The Euroqol-5D-5L (EQ-5D-5L) was employed [29]. This self-administered questionnaire is organized into two sections: (1) EQ-5D-5L index: A descriptive system that evaluates five dimensions: mobility, self-care, usual activities, pain/discomfort, and anxiety/depression. Each dimension has five levels according to its severity (no problems, slight problems, moderate problems, severe problems, and extreme problems). This is based on the response combination of the five dimensions and their degrees of severity and ranges from 1 (best health) to 0 (worst health). (2) A vertical visual analog scale (EQ-VAS) where participants indicate their health status, ranging from “the best health you can imagine” with a rating of 100 to “the worst health you can imagine” at 0 [29].

Mental health and mental well-being symptoms: The Strengths and Difficulties Questionnaire (SDQ) was used. It is composed of 25 items, which provide scores on 5 subscales: emotional symptoms, conduct problems, hyperactivity/inattention, peer relationship problems, and prosocial behavior (positive mental health). The first four refer to emotional and conduct difficulties (total rating), and the fifth refers to positive socialization behaviors. Each item is graded from 0 to 2 points according to the answers “Not true”, “Partly true”, or “Totally true”. The grading is inverse for positive characteristics. The total score ranges between 0 and 50 (no difficulty versus maximum difficulty); a 16-point cut-off is considered to represent a possible mental health alteration [30]. This questionnaire has been validated for adolescents aged 11 to 16 years with a 0.82 Cronbach Alpha for the total rating [31,32].

### 2.8. Analysis

The statistics program R version 4.3.2 was employed. A descriptive statistical analysis was carried out for the sociodemographic variables of the total sample stratified into the four groups at baseline. The categorical variables were summarized by means of frequencies and percentages, while mean and standard deviation (SD) were used for the numerical variables. The baseline scores of the SDQ, the EQ-VAS, and the EQ5D-5L index of the IG were analyzed and showed a 95% confidence interval (CI). In order to identify the existence of significant differences among the various interventions for both the sociodemographic and clinical variables, an analysis of variance was performed for the numerical variables, and the chi-square test was performed for the categories.

Scores for the EQ-VAS and EQ5D-5L index were evaluated with mixed linear models considering interview, EC, and IG. Models were adjusted for gender, nationality, HDIpc, and baseline SDQ. Missing data were treated through multiple data imputations using the predictive means method, with ten imputations performed in each of the five iterations. For all the analyses, a value of *p* > 0.05 was required for statistical significance.

## 3. Results

**Figure 1:**
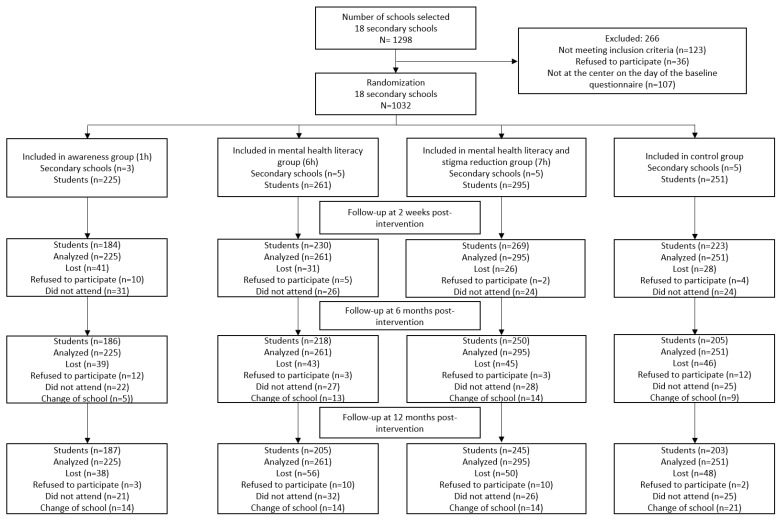
Participant flowchart.

Of the 1298 adolescents who agreed to participate, 1032 were finally included. Of the 266 who were excluded, 123 did not fulfill the inclusion criteria, 107 were absent on the day of data gathering, and 36 did not wish to participate (Figure 1).

Girls represented 49.6%, 79.1% of the male and female participants had Spanish nationality, and the mean age was 14.20 years (SD 0.58) 0.58). The most prevalent foreign nationalities were Chinese (9.7%), Filippino (6.48%), and Bolivians and Dominicans (both 6.01%). Most of the participants (46.2%) resided in neighborhoods with medium HDIpc levels, while 12.9% lived in areas with either a high or low HDIpc. Significant differences were observed among the IGs for both the distribution of nationality (*p* < 0.001) and HDIpc (*p* > 0.001). The G6h group presented a lower percentage of participants of foreign nationality, and the G1h had a smaller number of participants with a high HDIpc (Table 1).

The total score for the EQ-VAS was 77.84 (CI = 76.77–78.91) and 0.91 (CI = 0.90–0.92) for the EQ-5D-5L index at baseline. The mean score for the SDQ was 12.27 (CI = 11.95–12.59). Girls had significantly worse scores than boys in the EQ-5D-5L (EQ-VAS) and SDQ (*p* < 0.001) (Table 2).

When analyzing nationality, it can be observed that the participants of a foreign nationality presented worse scores in the EQ-VAS (75.6 (CI = 73.13–78.19; *p* = 0.039)) and the SDQ (13.30 (CI = 12.62–13.99; *p* < 0.001)) than the Spanish participants (78.42 (CI = 77.24–79.59) and 12.00 (CI = 11.63–12.36), respectively). With respect to HDIpc, individuals in the lowest category had better EQ-5D-5L index scores than those with a medium–high and high HDIpc (*p* = 0.008). No statistically significant differences were found for the EQ-VAS and the SDQ (Table 2).

With regard to the SDQ dimensions of gender and nationality, in addition to a greater total score, the female participants presented higher scores for “emotional symptoms” (4.00 (CI = 3.80–4.20)) and “prosocial behavior” (7.99 (CI = 7.84–8.14)) than the males (*p* < 0.001) (Table 3). In contrast, the students of a foreign nationality had higher scores in “conduct problems” (2.67 (CI = 2.43–2.91)) and “peer relationship problems” (2.49 (CI = 2.24–2.73)) than the Spanish ones (*p* < 0.05). Only in the “prosocial behavior” dimension was a greater score observed in the Spanish population, namely, 7.82 (CI = 7.71–7.94), compared to the foreign one, namely, 7.44 (7.19–7.70; *p* = 0.004). No statistically significant differences were found between the IGs and the CG for the SDQ total score.

Table 4 depicts the EQ-5D-5L scores at each follow-up. The mean baseline score of the EQ-VAS was 77.84 (CI = 76.77–78.91), with the G6h group presenting the highest result of 79.26 (CI = 77.14–81.37). In general terms, all the groups (IGs and GC) had improved EQ-VAS scores post-intervention, with the exception of G6h, which decreased to 78.19 (CI = 76.01–80.36). The tendency of scores to increase was not repeated at 6 months; all the groups dropped to below their baseline levels except the CG, which rose to 83.17 (CI = 80.9–85.39), a statistically significant difference. At 12 months, all the groups presented lower scores than at baseline. The G7h group had the highest score of 77.31 (CI = 75.05–79.58), which was statistically significant compared to the other groups (*p* < 0.001).

The mean baseline EQ5D-5L index was 0.91 (CI = 0.90–0.92) and was similar between the groups (Table 4). Post-intervention, the CG and G7h scores stayed the same, while there was a decrease in the G1h and G6h groups (*p* < 0.01). At six months, the observed trends in the EQ-5D-5L index reversed, and an improvement in G1h and G6h and a decrease in the other groups with respect to the prior evaluation were reported. Despite this change, mean scores were lower than at baseline, and the CG had the highest score with 0.91 (CI = 0.90–0.93). All the groups exhibited reduced scores one year after study commencement.

## 4. Discussion

Our study describes the relationship between the effectiveness of an MHL program with three interventions of differing intensity and QOL in a sample of adolescents from Barcelona (Spain). It also assesses the participants’ degree of QOL and mental well-being with respect to their socioeconomic/sociodemographic variables. The findings demonstrate that while MHL-based interventions had a significant impact on the adolescents’ QOL, there are temporal limitations, and socioeconomic/sociodemographic factors are linked to both QOL and mental well-being.

The SDQ baseline results show that the global scores were higher for the girls than the boys, agreeing with the findings of Ortuño et al. [33] but not, however, with the Spanish National Health Study [34]. When contrasting the different dimensions of the questionnaire according to gender with the current literature, results vary. In our study, the girls presented significantly higher scores in the “emotional symptoms” and “prosocial behavior” dimensions, while in the National Study, the boys scored higher in “conduct problems”, “hyperactivity/inattention”, and “peer relationship problems”.

In our study, all SDQ scores were higher in girls than in boys, while in the Spanish National Health Study, boys had higher scores in all dimensions except for emotional symptoms [34].

It was also observed that the participants of a foreign nationality had lower scores in “conduct problems” and “peer relationship problems” than the Spanish adolescents who had higher scores in “prosocial behavior”. Such findings concur with the National Study: no significant differences were obtained for the SDQ scores related to HDIpc [34]. We found statistically significant differences in QOL according to gender, nationality, and HDIpc. In general, the male participants presented a greater QOL score than the females, which might reflect gender differences when perceiving and scoring QOL. This is in accordance with patterns that have been observed by other authors, such as Mikkelsen [35] and Hadianfard [36], at the international level, as well as national-level observations [37].

Regarding nationality, we observed that results from the FRESC study carried out in Barcelona showed that subjects from other countries presented lower self-rated health than Spanish ones [3]. Another study performed in Spain reported differences between migrant and native populations regarding QOL [38], and in the neighboring country of Portugal, Botelho et al. also observed the same trend in the QOL of foreign adolescents and young adults [39]. This partially concurs with our findings, although they were only statistically significant for the EQ-VAS. These differences could be explained by possible socio-cultural barriers, situations of discrimination, or racism in a life stage where acceptance is sought at the group and social levels.

It is also noteworthy that we observed QOL differences related to socioeconomic status but only for the EQ-5D-5L index. These findings differ from the literature, in which a greater socioeconomic family status is associated with better QOL in adolescents [38,39,40,41]; Kim Kw et al. explain that “this socioeconomic status may produce an impact on QOL at the beginning of adolescence, but in the long term is reduced over time” [41]. These factors may be due to the fact that as they have more economic resources, they can access resources that help promote better physical and mental well-being and better nutrition, and their basic needs are satisfied.

Our study interventions improved adolescent QOL in the short term, but this was not maintained over time. Indeed, at the six-month evaluation, the CG had a better EQ-VAS score than the IGs. Such an increase could be due to other interventions in the school environment that the research team could not control or external factors leading to better QOL self-perception for the CG.

At twelve months, all the scores for the EQ5D-5L index and the EQ-VAS had decreased compared to the baseline. These results are in line with studies on adults, such as those conducted by Ben et al. [42], in which short-term changes were reported, but changes at six and twelve months were not reported. Lawrence et al. [43] also found no significant changes in QOL after a similar intervention. We observed that the most extensive intervention (G7h) presented the best EQ-VAS score at twelve months post-intervention compared to the other two groups (G1h and G6h). The participation of an individual with a mental disorder might have led to a positive impact on the subjects’ QOL despite the fact that stigma reduction was not achieved [22]. It appears that reinforcing sessions with a greater component of stigma reduction are required to maintain results over time.

### Limitations

Our study has some limitations. Firstly, it was performed in Barcelona, which may limit its extrapolation to the rest of the Spanish adolescent population. Nevertheless, the participating schools were located in all districts of the city and represented the diversity of its educational system. Secondly, variations in the implementation of the three interventions in the schools could have influenced our results. Despite employing validated tools to evaluate QOL, the results were based on self-reported data, which might have produced a response bias. Thirdly, as it was impossible to carry out all the interventions and evaluations simultaneously, they were performed on different days throughout the year. Such time differences could have influenced our results, for instance, depending on whether the adolescents were sitting exams or beginning the holiday period, and these should be considered variables in the future.

## 5. Conclusions

A mental health literacy program with a stigma reduction component conducted over a longer period could improve the short-term quality of life in adolescents. Female participants and those of a foreign nationality presented worse quality of life and mental well-being scores. Further studies are required to evaluate the different contents and duration of mental health literacy interventions. In addition, it is important to take into consideration gender and nationality as factors related to quality of life and mental well-being in adolescents.

## Figures and Tables

**Table 1:** Sociodemographic characteristics of the sample.

	CG (*n* = 251)		IG 1 h (*n* = 225)		IG 6 h (*n* = 261)		IG 7 h (*n* = 295)		Total (*n* = 1032)		*p*
	*n*	%	*n*	%	*n*	%	*n*	%	*n*	%	
Gender											0.570
Female	127	50.6%	118	52.4%	130	49.8%	137	46.4%	512	49.6%	
Male	124	49.4%	107	47.6%	131	50.2%	158	53.6%	520	50.4%	
Nationality											
Spanish	194	77.3%	156	69.3%	225	86.2%	241	81.7%	816	79.1%	<0.001
Foreign	57	22.7%	69	30.7%	36	13.8%	54	18.3%	216	20.9%	
Income level *											
Low	46	18.3%	16	7.1%	46	17.6%	25	8.5%	133	12.9%	<0.001
Medium–Low	38	15.1%	41	18.2%	54	20.7%	58	19.7%	191	18.5%	
Medium	100	39.8%	153	68.0%	97	37.2%	137	46.4%	487	47.2%	
Medium–High	29	11.6%	13	5.8%	30	11.5%	16	5.4%	88	8.5%	
High	38	15.1%	2	0.9%	34	13.0%	59	20.0%	133	12.9%	
Age (mean and standard deviation)	14.25	0.57	14.17	0.57	14.21	0.64	14.16	0.54	14.20	0.58	0.25

* Income level according to the HDIpc Index: low (<0.7), medium–low (0.7–0.89), medium (0.9–1.09), medium–high (1.1–1.29), and high (>1.3); CG: control group; IG: intervention group; *p*: *p*-value.

**Table 2:** Sociodemographic characteristics of participants at baseline and their relationship with quality of life and mental health.

	EuroQol-5D-5L VAS				EuroQol-5D-5L Basal Index				Strengths and Difficulties Questionnaire			
	Mean	CI Min	CI Max	*p*	Mean	CI Min	CI Max	*p*	Mean	CI Min	CI Max	*p*
Total Score	77.84	76.77	78.91		0.91	0.90	0.92		12.27	11.95	12.59	
Gender				<0.001				<0.001				<0.001
Female	74.86	73.22	76.5		0.90	0.89	0.91		12.85	12.38	13.32	
Male	80.78	79.45	82.11		0.93	0.92	0.94		11.70	11.26	12.14	
Nationality				0.039				0.335				0.001
Spanish	78.42	77.24	79.59		0.91	0.91	0.92		12.00	11.63	12.36	
Foreign	75.66	73.13	78.19		0.90	0.89	0.92		13.30	12.62	13.99	
Income level *				0.549				0.008				0.28
Low	77.21	74.31	80.11		0.93	0.91	0.94		11.96	11.04	12.89	
Medium–Low	78.42	75.78	81.06		0.92	0.90	0.94		12.03	11.3	12.75	
Medium	78.51	76.97	80.06		0.91	0.90	0.92		12.27	11.78	12.76	
Medium–High	75.72	72.59	78.84		0.87	0.84	0.90		13.42	12.32	14.53	
High	76.60	73.37	79.83		0.91	0.89	0.93		12.17	11.37	12.98	

* Income level according to the HDIpc index: low (<0.7), medium–low(0.7–0.89), medium (0.9–1.09), medium–high (1.1–1.29), and high (>1.3); EuroQol-5D-5L VAS: visual analog scale with score range 0–100; EuroQol-5D-5L Basal Index: score range 0–1.0; Strengths and Difficulties Questionnaire: total score from 0 (no difficulty) to 50 (maximum difficulty); CI: 95% confidence interval; Min: minimum; Max: Maximum; *p*: *p*-value.

**Table 3:** Strengths and Difficulties Questionnaire (SDQ) scores by gender and nationality at baseline.

	Male			Female			
SDQ Dimensions	Mean	CI Min	CI Max	Mean	CI Min	CI Max	*p*
Hyperactivity/Inattention	4.59	4.39	4.78	4.65	4.46	4.84	0.669
Emotional Symptoms	2.99	2.83	3.15	4.00	3.80	4.20	<0.001
Conduct Problems	2.32	2.17	2.46	2.37	2.22	2.52	0.618
Peer Problems	1.80	1.66	1.95	1.83	1.69	1.98	0.776
Prosocial Behavior	7.51	7.36	7.65	7.99	7.84	8.14	<0.001
Emotional and Behavioral Difficulties	11.7	11.26	12.14	12.85	12.38	13.32	<0.001
	**Spanish Nationality**			**Foreign Nationality**			
**SDQ Dimensions**	**Mean**	**CI Min**	**CI Max**	**Mean**	**CI Min**	**CI Max**	***p***
Hyperactivity/Inattention	4.66	4.50	4.82	4.47	4.21	4.74	0.284
Emotional Symptoms	3.44	3.30	3.59	3.67	3.37	3.97	0.17
Conduct Problems	2.25	2.14	2.37	2.67	2.43	2.91	0.001
Peer Problems	1.64	1.53	1.75	2.49	2.24	2.73	<0.001
Prosocial Behavior	7.82	7.71	7.94	7.44	7.19	7.70	0.004
Emotional and Behavioral Difficulties	12.00	11.63	12.36	13.3	12.62	13.99	0.001
**SDQ Dimensions**	**Mean**		**CI Min**		**CI Max**		**Total (*n*)**
Hyperactivity/Inattention	4.62		4.48		4.76		1032
Emotional Symptoms	3.49		3.36		3.62		
Conduct Problems	2.34		2.24		2.44		
Peer Problems	1.82		1.71		1.92		
Prosocial Behavior	7.75		7.64		7.85		
Emotional and Behavioral Difficulties	12.27		11.95		12.59		

Dimensions “Strengths and Difficulties Questionnaire” (SDQ): each scale presents a score from 0 (no difficulty) to 10 (maximum difficulty); emotional and behavioral difficulties: total score from 0 (no difficulty) to 50 (maximum difficulty); CI: 95% confidence interval; Min: minimum; Max: Maximum; *p*: *p*-value.

**Table 4:** Changes in quality of life (VAS and Index) in relation to the degree of mental health literacy intervention over time.

	CG (*n* = 251)			IG 1 h (*n* = 225)			IG 6 h (*n* = 261)			IG 7 h (*n* = 295)			Total (*n* = 1032)			*p*
Time	Mean	CI Min	CI Max	Mean	CI Min	CI Max	Mean	CI Min	CI Max	Mean	CI Min	CI Max	Mean	CI Min	CI Max	
	**EuroQol-5D-5L VAS**															
Basal	75.44	73.26	77.62	78.74	76.55	80.92	79.26	77.14	81.37	77.95	75.88	80.03	77.84	76.77	78.91	0.079
Post	76.71	74.31	79.11	79.53	77.09	81.97	78.19	76.01	80.36	81.13	79.05	83.21	78.96	77.83	80.09	0.038
6 months	83.17	80.95	85.39	73.22	70.01	76.43	77.96	75.52	80.40	72.40	69.23	75.57	76.60	75.18	78.03	<0.001
1 year	67.69	63.52	71.85	65.56	61.34	69.77	67.53	63.70	71.36	77.31	75.05	79.58	69.93	68.12	71.74	<0.001
	**EuroQol-5D-5L Index**															
Basal	0.92	0.90	0.93	0.91	0.89	0.92	0.91	0.90	0.93	0.91	0.89	0.92	0.91	0.90	0.92	0.930
Post	0.92	0.90	0.93	0.87	0.85	0.89	0.87	0.84	0.89	0.91	0.90	0.92	0.89	0.88	0.90	<0.01
6 months	0.91	0.90	0.93	0.88	0.87	0.90	0.90	0.88	0.91	0.88	0.86	0.90	0.89	0.88	0.90	0.069
1 year	0.87	0.84	0.89	0.87	0.85	0.90	0.87	0.85	0.89	0.83	0.80	0.86	0.86	0.85	0.87	0.057

EuroQol-5D-5L VAS: visual analog scale with score range 0–100; EuroQol-5D-5L Index: score range 0–1; Euroqol 5D-5L controlled for gender, nationality, socioeconomic level, and total baseline Strengths and Difficulties Questionnaire (SDQ) (intervention–control); CI: 95% confidence interval; Min: minimum; Max: maximum; *p*: *p*-value adjusted for time, gender, nationality, income level, and Strengths and Difficulties Questionnaire (SDQ); CG: control group; IG: intervention group.

## Data Availability

The original contributions presented in this study are included in the article. Further inquiries can be directed to the corresponding author.
